# Supplementary figures and images for: Exploring the immune responses triggered by vaccine formulations containing the recombinant Schistosoma mansoni 14kDa fatty acid-binding protein
Source: PLoS One. 2025 Dec 8;20(12):e0338310. doi: 10.1371/journal.pone.0338310 (PMC12685172; doi:10.1371/journal.pone.0338310)

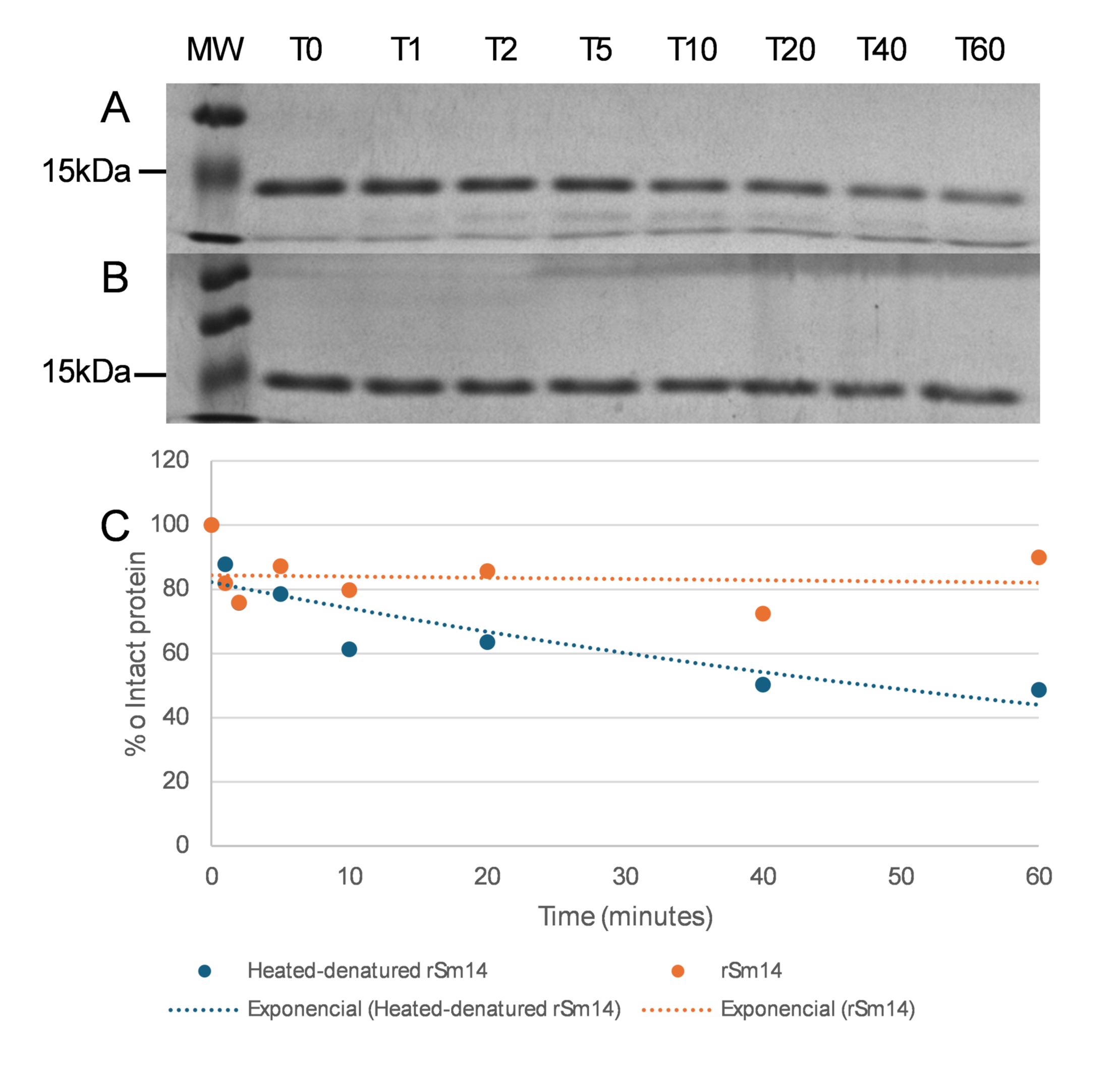

Supplement: S2 Fig — Heat-denatured rSm14 (A) and rSm14 (B) were subjected to trypsin digestion at 25°C for 0 (T0), 1 (T1), 2 (T2), 5 (T5), 10 (T10), 20 (T20), 40 (T40), and 60 (T60) minutes. The degradation profiles were analyzed by 15% SDS-PAGE and visualized with Coomassie Brilliant Blue G-250 (A–B). Molecular weight markers (MW) used were Precision Plus Protein™ Dual Color Standards (Bio-Rad). Densitometric analysis of the intact rSm14 band over time was performed using ImageJ (C). T0 values were used for normalization, and results are expressed as the percentage of intact protein with the Exponential trendline. (TIF) [file pone.0338310.s002.tif]

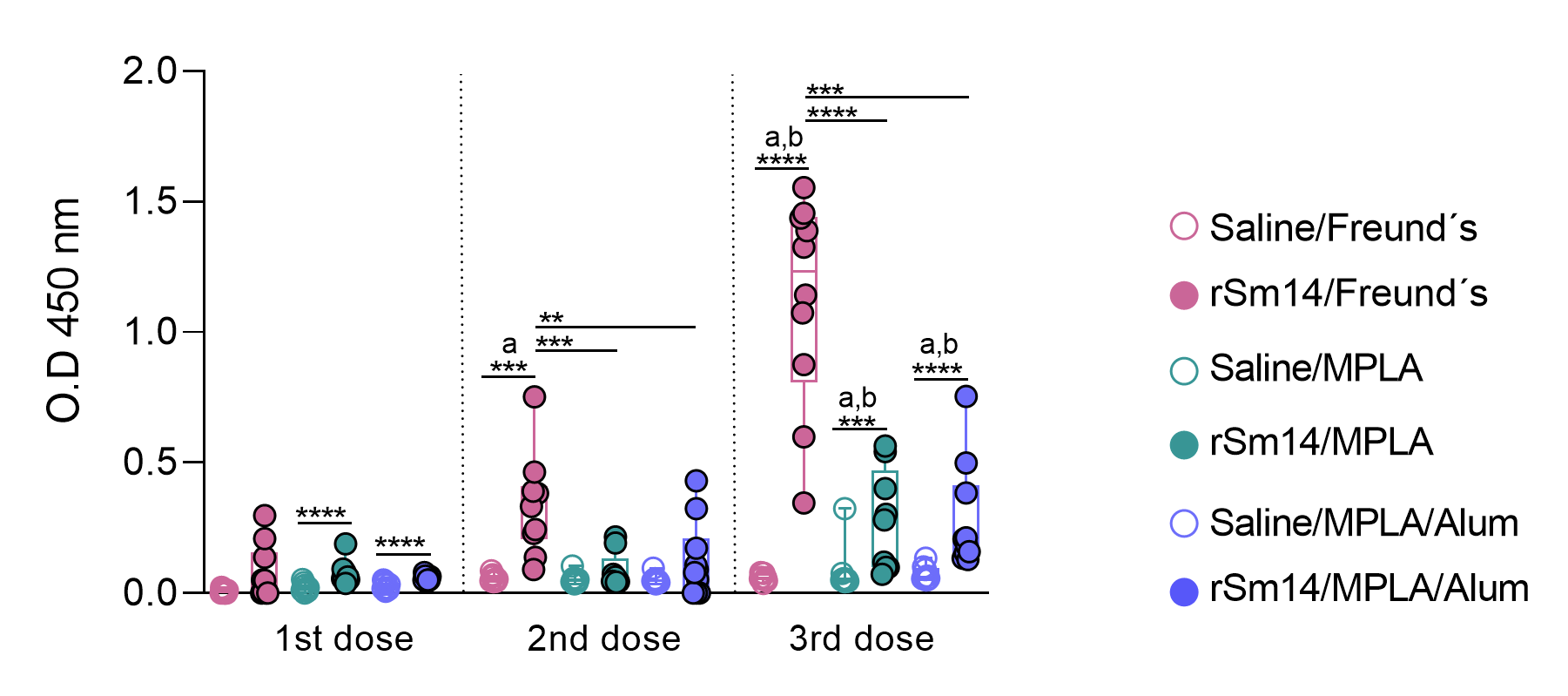

Supplement: S3 Fig — Sera from mice immunized with rSm14 were obtained 15 days after each vaccine dose to evaluate the recognition of SWAP. Box and whiskers represent the distribution of IgG antibody levels in different sera. Each symbol represents an animal (9–10 mice per group). Differences were determined by two-way ANOVA followed by Tukey’s multiple comparisons test. The letters “a” and “b” denote statistically significant differences in relation to the first and second immunization doses, respectively. (TIF) [file pone.0338310.s003.tif]
