# Supplementary material for: Exploring the immune responses triggered by vaccine formulations containing the recombinant Schistosoma mansoni 14kDa fatty acid-binding protein
Source: PLoS One. 2025 Dec 8;20(12):e0338310. doi: 10.1371/journal.pone.0338310 (PMC12685172; doi:10.1371/journal.pone.0338310)
Supplement: S1 Table — (DOCX) [file pone.0338310.s007.docx]

| **Supplementary Table 1: Identification of proteins by mass spectrometry.** | | | | | |
| --- | --- | --- | --- | --- | --- |
| **Band** | **Accession number** | **Name** | **Coverage (%)** | **Number of peptides** | **Molecular**  **weight (Da)** |
| 1 | Smp_095360.1 | 14 kDa fatty acid-binding protein | 38 | 5 | 14,848 |
| 2 | Smp_095360.1 | 14 kDa fatty acid-binding protein | 19 | 2 | 14,848 |
