# Supplementary material for: Exploring the immune responses triggered by vaccine formulations containing the recombinant Schistosoma mansoni 14kDa fatty acid-binding protein
Source: PLoS One. 2025 Dec 8;20(12):e0338310. doi: 10.1371/journal.pone.0338310 (PMC12685172; doi:10.1371/journal.pone.0338310)
Supplement: S2 Table — (DOCX) [file pone.0338310.s008.docx]

**Supplementary Table 2:** Summary of the cellular immune response triggered by the different formulations after the third dose of immunization.

|  | rSm14/Freund’s | rSm14/MPLA | rSm14/MPLA/Alum |
| --- | --- | --- | --- |
|  |  |  |  |
| **Cell types** | | | |
| CD4^+^ T cells | NS | NS | NS |
| Effector CD4^+^ T cells | **↑**2.7x; **↑**2.0x | **↑**1.4x | **↑**1.2x; **↑**1,7x |
| EM CD4^+^ T cells | **↓**1.3x | ↓1.4x | **↑**1,3x; **↑**1.2x |
| CM CD4^+^ T cells | **↓**3.2x | **↑**2.0x; **↑**1.4x | **↓**3.1x; **↓**3.6x |
| CD8^+^ T cells | NS | **↓**1.1x | NS |
| Effector CD8^+^ T cells | **↑**2.0x | NS | **↑**1.8x |
| EM CD8^+^ T cells | NS | NS | **↑**1.20x; **↑**1.6x |
| CM CD8^+^ T cells | NS | NS | **↓**2.8x |
| CD19^+^ cells | **↓**1.8x | **↓**1.5x | **↑**1.2x |
| CD19^+^CD27^+^ cells | **↑**2.3x; **↑**2.6x; **↑**1.5x | **↑**1.7x | **↑**2.0x; **↑**1.9x |
| **Cytokines** | | | |
| TNF | **↑**2.3x; **↑**2.0x; **↑**1.1x  ↑11.5x **↑**5.5x | NS | NS |
| IFN | **↑**1.9x; **↑**2.3x  ↑5.2x; **↑**3.3x | NS | NS |
| IL-6 | **↑**3.4x  ↑3.8x; ↑1.6x; **↑**2.4x | NS | **↑**4.0x  **↑**2.3x |
| **IgG and subclasses** | | | |
| IgG | **↑**58x;**↑**1.7x; **↑**8.0x  ↑2.4x; **↑**1.6x | **↑**110.6x; **↑**21x; **↑**165.5x | **↑**92.6x; **↑**3x; **↑**192,2x |
| IgG1 | **↑**16.1x; **↑**2.7x; **↑**261.3x  ↑2.8x | **↑**107.3x; **↑**5x; **↑**461,1x | **↑**11.2x; **↑**2x; **↑**388,7x  **↑**3.3x |
| IgG2c | **↑**74.4x; **↑**4.6x; **↑**248.3  ↑16.8x; **↑**55.3x | NS | NS |

NS (not significant); EM (effector memory); CM (central memory). Green and red arrows indicate significant differences compared to the first and second doses, respectively. Blue arrows indicate differences between the immunized group and the saline control. Black, pink, and purple arrows indicate differences between the rSm14/Freund and rSm14/MPLA groups, rSm14/Freund and rSm14/MPLA/Alum groups, and rSm14/MPLA/Alum and rSm14/MPLA groups, respectively.
