# Supplementary material for: Exploring the immune responses triggered by vaccine formulations containing the recombinant Schistosoma mansoni 14kDa fatty acid-binding protein
Source: PLoS One. 2025 Dec 8;20(12):e0338310. doi: 10.1371/journal.pone.0338310 (PMC12685172; doi:10.1371/journal.pone.0338310)
Supplement: S1 File — Raw Images of the 15% SDS-PAGE (C) and Western blot (D) using a monoclonal 6x-His-tag antibody for the analysis of rSm14 expression and purification presented in Fig 1 – Molecular weight markers (lane 1); non-IPTG induced (lane 2); 1mM IPTG-induced protein expression in bacterial culture (lane 3); bacterial lysate under denaturing conditions (lane 4); and purified rSm14 fraction (lane 5). Raw image of the SDS-PAGE gels presented in S2 Fig Heat-denatured rSm14 (A) and rSm14 (B) subjected to trypsin digestion at 25°C for 0 (T0), (lane 2); 1 (T1) (lane 3); 2 (T2) (lane 4); 5 (T5) (lane 5); 10 (T10) (lane 6); 20 (T20) (lane 7); 40 (T40) (lane 8); and 60 (T60) minutes (lane 9); Molecular weight markers (lane 1). (PDF) [file pone.0338310.s009.pdf]

Figure 1: Expression and purification of the recombinant *Schistosoma mansoni* Sm14.

Panel C

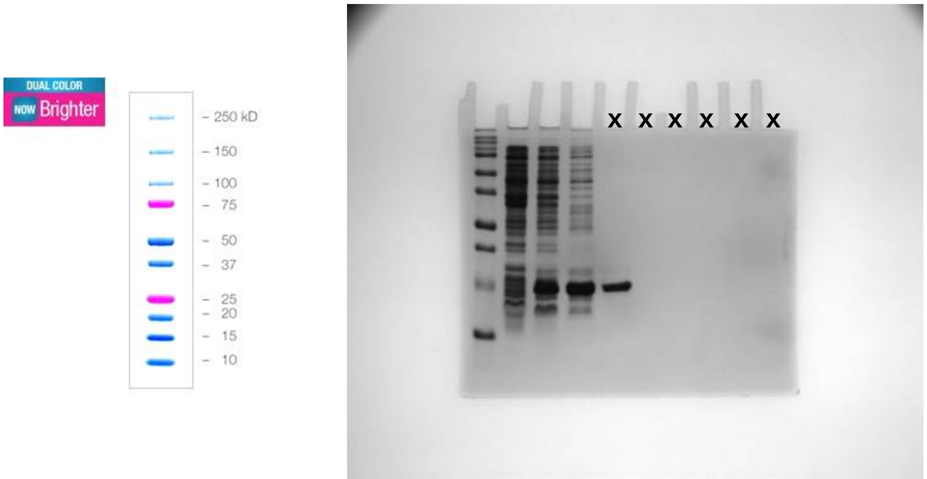

Lane 1: Molecular weight markers
Lane 2: non-IPTG induced
Lane 3: 1mM IPTG-induced protein expression in bacterial culture
Lane 4: bacterial lysate under denaturing conditions
Lane 5: purified rSm14 fraction

The image was captured using a photodocumentation system (VILBFR, DOC-PRINT VX5)

Panel D

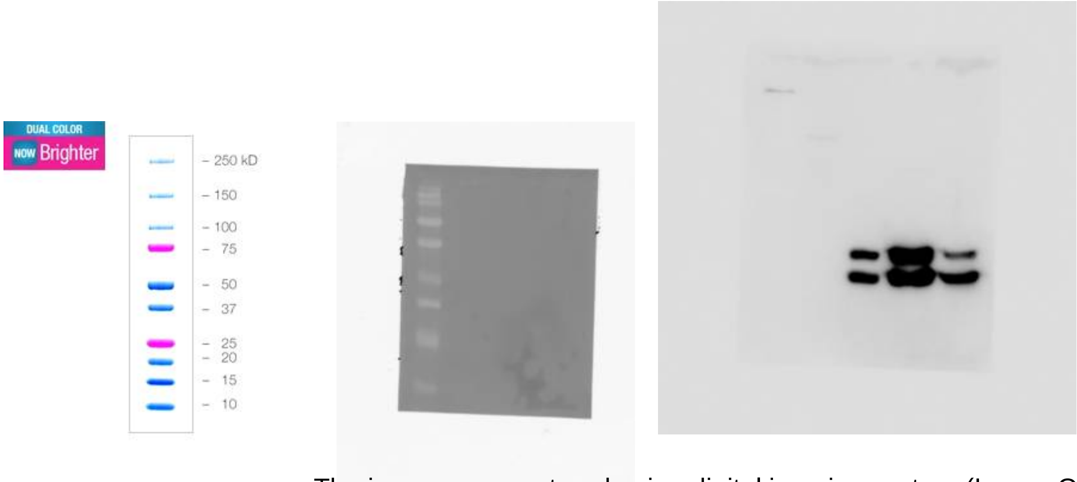

Lane 1: Molecular weight markers
Lane 2: non-IPTG induced
Lane 3: 1mM IPTG-induced protein expression in bacterial culture
Lane 4: bacterial lysate under denaturing conditions
Lane 5: purified rSm14 fraction

The image was captured using digital imaging system (Image Quant LAS 4000)

S2 Fig. Decay of intact rSm14 protein under trypsin proteolysis.

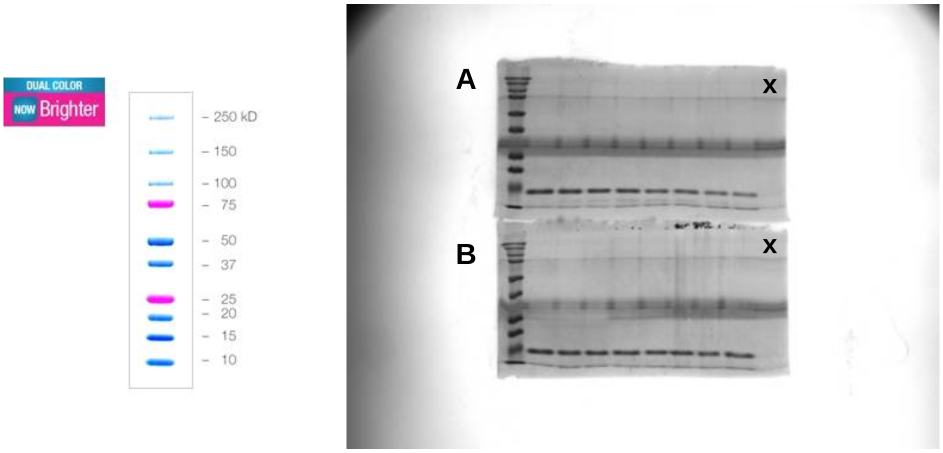

Heat-denatured rSm14 (A) and rSm14 (B) were subjected to trypsin digestion Trypsin digestion at 25°C for 0 (T0), (lane 2), 1 (T1) (lane 3), 2 (T2) (lane 4), 5 (T5) (lane 5), 10 (T10) (lane 6), 20 (T20) (lane 7), 40 (T40) (lane 8), and 60 (T60) minutes (lane 9). Lane 1: Molecular weight markers.

Both images were captured using a photodocumentation system (VILBFR, DOC-PRINT VX5)
