# Supplementary material for: Exploring the immune responses triggered by vaccine formulations containing the recombinant Schistosoma mansoni 14kDa fatty acid-binding protein
Source: PLoS One. 2025 Dec 8;20(12):e0338310. doi: 10.1371/journal.pone.0338310 (PMC12685172; doi:10.1371/journal.pone.0338310)
Supplement: Attachment — Submitted filename: response to referee 16092025.docx [file pone.0338310.s011.docx]

**Response to Referee’s comments PONE-D-25-18857**

**Dear Editor and Reviewers,**

**We appreciate the reviewers’ insightful comments and suggestions, which have helped improve the clarity and quality of our manuscript.**

**Please find below our point-by-point responses to each of the reviewers’ comments.**

Reviewer #1: Please, spell MPLA and explain EM: Effector memory?

**Response: MPLA spell was included in the abstract, page 1, line 18. EM stands for Effector memory, this information is included in the text at page 12, line 250**

Supplementary Figures 1-5 will be invaluable in the article.

**Response: We greatly appreciate the reviewer’s thoughtful suggestion. While we recognize the value of including these figures in the main text, we have opted to limit the number of figures. Considering that the data are already presented in the manuscript in a different format, we have chosen to keep them as supplementary figures. We believe this approach preserves the clarity and conciseness of the main manuscript while still making the data fully accessible to readers.**

Please devise a Table to summarize, abstract, compare, and contrast the final pre-challenge immune responses achieved following vaccination with the three different Sm14 formulations in order to clarify, tabulate, and emphasize all that is non-protective even following challenge with 30 cercariae.

**Response: We appreciate the reviewer's suggestion. Indeed, summarizing the findings related to the immune response elicited by the vaccine formulations after the third dose is a strategy that enhances the overall understanding of the results. Instead of presenting the data in a table, we chose to do so in a more visual manner through a graphical abstract, which has been included as Figure 7 in the article.**

Reviewer #2: Major Comments:
1. Lack of Protective Efficacy Despite Strong Immune Response:
• Although robust immune responses (e.g., IFN-γ, TNF, IgG2c, memory T/B cells) were induced, none of the vaccine formulations resulted in significant reduction in worm burden or egg count. This raises serious concerns about the functional relevance of these immune responses.

**Response: That is precisely the point we intended to highlight with our manuscript. As discussed in the manuscript (page 20, lines 445-449), the production of IgG antibodies and subclasses, as well as cytokines such as IFN-γ, following immunizations, is currently being assessed in Phase 2 clinical trials using rSm14 as the antigen. Although Phase 2 trials do not allow a direct correlation between resistance and immunogenicity, the authors suggest that the production of these factors could be associated with resistance to infection, as this association has been demonstrated in preclinical studies. However, it is important to note that in preclinical studies, a different form of rSm14 (fused with MBP) was used as the antigen.**

• The discussion hypothesizes potential causes (e.g., improper folding of rSm14 due to denaturation), but these claims remain speculative. The authors should consider validating this by testing conformational integrity (e.g., circular dichroism, native PAGE, or proteolytic sensitivity assays) or referencing studies that confirm folding status under their production conditions.

**Response: We thank the referee for the suggestion. Although it was not possible to perform circular dichroism, we conducted a proteolysis susceptibility assay to assess the protein folding. The results, included on page 10-11, lines 218 to 220, demonstrate that the protein is less susceptible to proteolysis than its heat-denatured form. These findings suggest the acquisition of a stable structure with fewer exposed cleavage sites. Together with the data showing recognition of the native protein in SWAP by anti-rSm14 antibodies, there is evidence that the protein’s conformational structure is correct; however, additional experiments would be needed to confirm this. These details have been added to the manuscript page 22 lines 479-487**.

2. Use of Freund’s Adjuvant in Vaccine Trials:
• While Freund’s adjuvant is commonly used in experimental settings, it is not suitable for clinical translation due to safety concerns. The authors acknowledge this but still heavily rely on data from Freund’s-formulated groups. Greater emphasis should be placed on clinically relevant adjuvants like MPLA/Alum.

**Response: As suggested by referee,** **we reduced the focus given to Freund's adjuvant in the manuscript**

3. Statistical Power and Interpretation:
• The study uses groups of 10 mice (with pooled blood samples for some flow cytometry analyses). While this is acceptable, individual-level data (e.g., scatter plots) for parasite burden and egg counts would strengthen the analysis and transparency of variance.

**Response**: **The use of pooled blood samples was necessary to obtain the number of cells required for the flow cytometry experiments, whereas parasite and egg counts were analyzed at the individual level. As suggested by the referee, we replaced Table 2 by Figure 6, which shows scatter plots of these results.**

• The p-values for worm and egg burden are generally >0.05, yet the manuscript refers to “trends.” PLOS ONE requires data to support claims robustly—avoid overinterpreting nonsignificant results.

**Response: We changed the description of these results**. **Additionally, we removed from the manuscript all text that was overinterpreting nonsignificant results.**

4. Antibody Functionality Not Adequately Addressed:
• Although anti-rSm14 antibodies recognized SWAP, it is unclear if these antibodies are functionally protective (e.g., neutralizing, opsonizing, complement-fixing). A functional assay such as in vitro parasite killing or opsonophagocytosis would be valuable, or at least a clearer discussion of this limitation.

**Response: As suggested, we highlighted the limitation of not having assessed the protective functionality of the antibodies raised against Sm14 in page 22 lines 483-486.**

5. Ethics and Methodology Reporting:
• Ethical approval is mentioned appropriately (license numbers provided).
• However, key methodological details—such as the justification for 30 vs. 100 cercariae challenge in different trials, and reproducibility between batches—need clarification.

**Response: As suggested, we included the justification of using 30 and 100 cercariae challenge in different trials page 17 lines 389-392 . Regarding protein batch, the same batch was used in all immunization trials, this information was added to the manuscript, page 6 line 109.**

Minor Comments:
• Typographical and Language Issues:
• Example: Line 16 – “described” should be “describe.” - **done**
• Line 430 – “native ,” spacing error – **done**
• Please review the manuscript thoroughly for minor typographic inconsistencies -**done**
• Figures & Supplementary Files:
• Ensure that all figure legends are complete and informative - **done**
• High-resolution images and fully labeled flow cytometry panels are critical for peer review. Please double-check Supplementary Figures S1–S5 for completeness - **done**
• Data Availability Statement:
• Authors claim all data are available within the manuscript and supplements. Please ensure raw flow cytometry data or ELISA plate readers are available upon request or in a public repository as per PLOS Data Policy **- raw flow cytometry data and Elisa plate readers are in the ArcaDados public repository under** [**https://doi.org/10.35078/BADAEY**](https://doi.org/10.35078/BADAEY)**. This information was added in the manuscript**

Regarding journals requirements, please find below the checklist and comments

**1. Please ensure that your manuscript meets PLOS ONE's style requirements, including those for file naming. The PLOS ONE style templates can be found at**

[**https://journals.plos.org/plosone/s/file?id=wjVg/PLOSOne_formatting_sample_main_body.pdf**](https://journals.plos.org/plosone/s/file?id=wjVg/PLOSOne_formatting_sample_main_body.pdf) **and**

[**https://journals.plos.org/plosone/s/file?id=ba62/PLOSOne_formatting_sample_title_authors_affiliations.pdf**](https://journals.plos.org/plosone/s/file?id=ba62/PLOSOne_formatting_sample_title_authors_affiliations.pdf) **– checked and corrected when needed**

**2. Thank you for stating the following financial disclosure:**

**“This study was financed in part by the Coordination for the Improvement of Higher Education Personnel (Coordenação de Aperfeiçoamento de Pessoal de Nível Superior-CAPES) – Finance Code 001, Instituto René Rachou, Conselho Nacional de Desenvolvimento Científico e Tecnológico–Brasil (Grant Nos. 306188/2022-8 and 315540/2023-0), Rede De Pesquisa em Doenças Infecciosas Humanas e Animais do Estado de Minas (RED00313-16), Rede Mineira de  Imunobiológicos (RED00067-23), Rede Mineira de Investigação em Mucosas e Pele (RED – 00096-22) and Programa de Pós-Graduação em Ciências da Saúde – IRR.”**

**Please state what role the funders took in the study.  If the funders had no role, please state: "The funders had no role in study design, data collection and analysis, decision to publish, or preparation of the manuscript."**

**Response: new statement was included with the information above as follows: “This study was financed in part by the Coordination for the Improvement of Higher Education Personnel (Coordenação de Aperfeiçoamento de Pessoal de Nível Superior-CAPES) – Finance Code 001, Conselho Nacional de Desenvolvimento Científico e Tecnológico–Brasil (Grant Nos. 306188/2022-8 and 315540/2023-0), FAPEMIG - Rede De Pesquisa em Doenças Infecciosas Humanas e Animais do Estado de Minas (RED00313-16), Fapemig - Rede Mineira de  Imunobiológicos (RED00067-23), Fapemig - Rede Mineira de Investigação em Mucosas e Pele (RED – 00096-22) and FAPEMIG (APQ-03113-24, APQ-02481-23). The funders had no role in study design, data collection and analysis, decision to publish, or preparation of the manuscript ”.**

**If this statement is not correct you must amend it as needed – done**

**Please include this amended Role of Funder statement in your cover letter; we will change the online submission form on your behalf - done**

**3. PLOS ONE now requires that authors provide the original uncropped and unadjusted images underlying all blot or gel results reported in a submission’s figures or Supporting Information files. This policy and the journal’s other requirements for blot/gel reporting and figure preparation are described in detail at** [**https://journals.plos.org/plosone/s/figures#loc-blot-and-gel-reporting-requirements**](https://journals.plos.org/plosone/s/figures#loc-blot-and-gel-reporting-requirements) **and** [**https://journals.plos.org/plosone/s/figures#loc-preparing-figures-from-image-files**](https://journals.plos.org/plosone/s/figures#loc-preparing-figures-from-image-files)**. When you submit your revised manuscript, please ensure that your figures adhere fully to these guidelines and provide the original underlying images for all blot or gel data reported in your submission. See the following link for instructions on providing the original image data:** [**https://journals.plos.org/plosone/s/figures#loc-original-images-for-blots-and-gels**](https://journals.plos.org/plosone/s/figures#loc-original-images-for-blots-and-gels)**.**

**In your cover letter, please note whether your blot/gel image data are in Supporting Information or posted at a public data repository, provide the repository URL if relevant, and provide specific details as to which raw blot/gel images, if any, are not available. Email us at** [**plosone@plos.org**](mailto:plosone@plos.org) **if you have any questions. – The original images of gels and Western blots are provided as supporting material; this information was included in the cover letter.**

**4. We notice that your supplementary table 1 is included in the manuscript file. Please remove them and upload them with the file type 'Supporting Information'. Please ensure that each Supporting Information file has a legend listed in the manuscript after the references list.- Supplementary Table 1 was removed from the manuscript**

**5. Please include captions for your Supporting Information files at the end of your manuscript, and update any in-text citations to match accordingly. Please see our Supporting Information guidelines for more information:** [**http://journals.plos.org/plosone/s/supporting-information**](http://journals.plos.org/plosone/s/supporting-information)**. - Done**

**If the reviewer comments include a recommendation to cite specific previously published works, please review and evaluate these publications to determine whether they are relevant and should be cited. There is no requirement to cite these works unless the editor has indicated otherwise. – not applicable.**
