# Supplementary material for: Exploring the immune responses triggered by vaccine formulations containing the recombinant Schistosoma mansoni 14kDa fatty acid-binding protein
Source: PLoS One. 2025 Dec 8;20(12):e0338310. doi: 10.1371/journal.pone.0338310 (PMC12685172; doi:10.1371/journal.pone.0338310)
Supplement: Attachment — Submitted filename: response to referee 14112025.docx [file pone.0338310.s012.docx]

**Response to Referee’s comments PONE-D-25-18857**

**Dear Editor and Reviewer,**

**Please find below our point-by-point responses to each of the reviewers’ comments.**

Major comments
1. Protein structural integrity – show proteolysis assay data
• The trypsin-digestion assay is a welcome addition. Please include a representative SDS-PAGE image or densitometric curve as a new supplementary figure to visually demonstrate stability of native rSm14 versus the heat-denatured control.

• Quantitative visualization would substantiate the conclusion that the protein retains conformational structure.
• (Reference: Ribeiro-Santos et al., Protein Expr Purif 2021, PMID 33743354.)

Response: I believe there might be a misunderstanding, as both the SDS-PAGE image and the corresponding densitometric curve were already included as Supplementary Figure 2 in the previous revision. Additionally, the raw SDS-PAGE images are provided in the “S1 Raw Images” file. We also tried to check the reference mentioned by the referee but could not locate it in PubMed — it may contain a typographical error, the PMID does not correspond to an article published in Protein Expr. Purif journal.

2. Antibody functionality discussion
• Since antibody functional assays (e.g., in vitro killing, complement activation, opsonophagocytosis) were not performed, please expand the Discussion with a brief paragraph describing how such assays could confirm protective relevance of anti-Sm14 antibodies in future studies.

• This addition would contextualize your acknowledgment of the limitation and strengthen translational interpretation.
• (See Hotez et al., Nat Rev Microbiol 2022, <https://doi.org/10.1038/s41579-022-00699-3>.)

Response: We attempted to retrieve the article mentioned by the reviewer, but it does not correspond to the doi or author plus journal plus year mentioned, it may contain a typographical error, nevertheless a new paragraph has been included in the Discussion (lines 418–424), citing studies that describe antibody-mediated immune mechanisms involved in vaccination-induced parasite killing, as well as the articles reporting these functional assays. In the case of Sm14, the precise mechanisms by which antibodies mediate parasite killing are not yet fully elucidated, and no specific functional assays have been developed so far.

New references were cited in this part of the manuscript

32.	Xu C ‐B, Verwaerde C, Grzych J ‐M, Fontaine J, Capron A. A monoclonal antibody blocking the Schistosoma mansoni 28‐kDa glutathione S‐transferase activity reduces female worm fecundity and egg viability. Eur J Immunol. 1991;21: 1801–1807.

33.	Grezel D, Capron M, Grzych J ‐M, Fontaine J, Lecocq J ‐P, Capron A. Protective immunity induced in rat schistosomiasis by a single dose of the Sm28GST recombinant antigen: Effector mechanisms involving IgE and IgA antibodies. Eur J Immunol. 1993;23: 454–460.

3. Summary of immune outcomes
• Figure 7 is an effective graphical abstract, but please add a concise table summarizing the main immune parameters (cytokines, antibody subclasses, CD4⁺/CD8⁺ T-cell memory, B-cell response, and protection outcome) for the three formulations.
• Such a table will help readers quickly compare formulations quantitatively.
• (Recommended by WOAH Terrestrial Manual 2024 Ch. 1.1.6 “Vaccine evaluation”).

Response: As requested by the referee, a summary table was included as Supplementary table 2 (S2 Table)

4. Statistical and methodological transparency
• Indicate exact p-values in figure legends or Supplementary Data (not only thresholds).

• Specify the number of biological replicates (n) per group and whether samples were pooled before analysis.

• These details align with PLOS ONE Statistical Reporting Guidelines 2025 (<https://journals.plos.org/plosone/s/statistical-reporting>).

Response: The exact p value was included in the figures. And the number of biological replicates (n) was added to each figure’s legend.

Minor comments
1. Abstract: Verify wording of line 18 — “formulated with either (i) MPLA, (ii) MPLA, or (iii) Freund’s adjuvant” appears to repeat MPLA. Likely one should be “MPLA/Alum.” Done
2. Introduction: Consider citing Riveau et al., Vaccine 2018 (https://doi.org/10.1016/j.vaccine.2018.03.016) regarding Bilharvax® to emphasize lessons on correlates of protection.

Response: The reference Riveau et al., Vaccine 2018 is already cited in line 66 of the introduction section.

3. Methods:
• Clarify whether the proteolysis assay was repeated (technical/biological replicates).

Response: The proteolysis assay was performed twice using the same protein batch, at 25 °C and 37 °C with similar results. This information was included in the manuscript (lines 131-132).

• Indicate detection limits (pg/mL) for each cytokine in the CBA assay.

Response: The detection limits for each cytokine in CBA were added to the manuscript.

4. Results:

• Provide geometric mean titers ± 95 % CI for antibody data if available.

Response: Antibody titer was performed with pooled samples

• Confirm all figure axes are labeled with units and sample size (n = ?).

Response: Sample size was included in the figure legend.

5. Discussion: The section on IL-6 and neutrophil recruitment could cite Lambrecht et al., Nat Rev Immunol 2024 (https://doi.org/10.1038/s41577-024-00928-8) for updated mechanisms of alum-induced IL-1β/IL-18 signaling.

Response: We thank the reviewer for this suggestion. We attempted to retrieve the cited article (Lambrecht et al., Nat Rev Immunol 2024; DOI: 10.1038/s41577-024-00928-8), but this DOI does not currently correspond to a published paper in Nature Reviews Immunology or in PubMed, suggesting a possible typographical error in the reference.

6. Language and style: A final proofreading pass is recommended (spacing before reference brackets, e.g., line 37 “public health [1]”). – Response: A final proofreading was performed.
